# Presence of Multiple Autoimmune Antibodies Involved in Concurrent Myositis and Myocarditis and Myasthenia Gravis Without Thymoma: A Case Report

**DOI:** 10.3389/fneur.2019.00770

**Published:** 2019-07-16

**Authors:** Zhijian Zhou, Xia Chen, Gonglu Liu, Jiali Pu, Jimin Wu

**Affiliations:** ^1^Department of Neurology, Affiliated Shaoxing Hospital of Traditional Chinese Medicine, Zhejiang Chinese Medical University, Shaoxing, China; ^2^Department of Neurology, Second Affiliated Hospital, College of Medicine, Zhejiang University, Hangzhou, China; ^3^Department of Neurology, Shangrao People's Hospital, Shangrao, China

**Keywords:** inflammatory myositis, myocarditis, myasthenia gravis, thymoma-associated antibodies, immunotherapy

## Abstract

Inflammatory myositis (IM) and myasthenia gravis (MG) are both immune disorders involving muscle. The concurrent presence of both conditions in the same patient is extremely rare and the diagnosis is important and challenging. Here, we report a case of concurrent myositis and myocarditis and MG without thymoma in a 69-year-old man with progressive proximal muscle weakness and dysphagia. As an atypical finding, the laboratory immunity assay showed the presence of multiple antibodies (acetylcholine receptor-Ab, titin-Ab, M7-Ab, smooth muscle alpha (SMA)-Ab, and citrate acid extract (CAE)-Ab). We predicted that thymoma-associated antibodies (titin-Ab, SMA-Ab, and CAE-Ab) and anti-M7 antibodies play an important role in the concurrent presence of MG and myositis and myocarditis. In this overlap case, immunotherapy was determined to be effective.

## Introduction

Myositis is an autoimmune disorder involving striated muscles. Myasthenia gravis (MG) is a well-characterized disease, generally mediated by autoantibodies targeting the acetylcholine receptor (AChR) or muscle-specific receptor tyrosine kinase (MuSK) at the neuromuscular junction (1). Although both are autoimmune disorders, concurrent MG and myositis is rare (2). Thymomas have a high frequency of autoimmune-associated disorders (45%), and 50% of patients with thymoma will develop MG. In addition 15% of MG patients have a thymoma (3).

The anti-mitochondrial antibody, anti-M7, is known to be involved in myocarditis of unknown etiology (4). Antibodies specific for titin, a large filamentous muscle protein that is essential for skeletal and heart muscle structure (5), as well as smooth muscle alpha (SMA) (6) and citrate acid extract (CAE) (7) from skeletal muscle, have been confirmed to be associated with thymoma-MG cases. Titin-Ab and another striational antibody, the ryanodine receptor (RyR)-Ab, have been detected in the rare patients with thymoma-MG and concurrent myositis (8). However, the pathogenicity of these two antibodies in MG-myositis remains to be confirmed.

Here, we present the case of a 69-year-old man with progressive proximal muscle weakness and dysphagia, diagnosed with MG without thymoma, myocarditis, and pathologically confirmed myositis. He also presented with multiple autoantibody-positive status for titin, M7, SMA, and CAE.

The patient explicitly agreed to his inclusion in this case report and gave written informed consent for publication.

## Case Presentation

A 69-year-old man was admitted to our hospital complaining of progressive and fluctuating proximal muscle weakness and dysarthria for 2 months. He presented with prominent fatigue and difficulty climbing stairs, as well as obvious weakness in holding his head up and chewing, but without ptosis. All the symptoms fluctuated during the day with dominant twilight activity. The patient reported a feeling of breathlessness in the anterior chest region. There was no family history of neurological disorders. On physical examination, a proximally accentuated muscle weakness was detected in all extremities (grade 4/5 MRC in arm abductors and hip flexors). No abnormal findings were detected in the remainder of the physical and neurological examinations. However, the neostigmine test was positive, with distinct improvements in both upper and lower limb fatigue as well as chewing and swallowing function.

**Figure 1:**
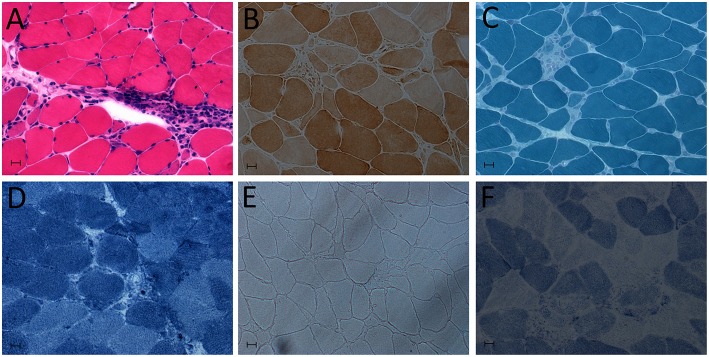
Deltoid muscle biopsy showing myositis. **(A)** Hematoxylin and eosin (HE) staining: abundant inflammatory cell infiltration of muscle. **(B)** Cytochrome c oxidase (COX) staining. **(C)** Modified Gomori trichrome (MGT). **(D)** NADH-tetrazolium reductase staining. **(E)** Oil Red O (OR) staining. **(F)** Succinate dehydrogenase (SDH) staining.

In terms of the laboratory examinations, serological tests showed abnormally increased levels of myocardial enzymes: creatine kinase (611 U/L), CK-MB (100 U/L) and cardiac troponin-T (cTnT; 1.580 ng/mL). Other routine laboratory tests revealed mostly normal values. Brain magnetic resonance imaging (MRI) showed no signs of cerebrovascular etiology of the presentation of dysarthria. The ultrasonic cardiogram showed an ejection fraction (EF) of 70%, with a slightly enlarged left atrium, aortic sinus expansion and aortic valve regurgitation. Normal myocardial thickness, coordinated activity, slight tricuspid regurgitation and left ventricular diastolic dysfunction were also observed. Electrocardiography showed atrial fibrillation, left axis deviation, suspicious Q wave of anterior intervertebral wall and ST-T changes. EMG findings also confirmed non-irritable myopathy. Fibrillation (++), positive sharpness (+), amplitude of 3.9 mV, time-limit shortened by 30.6%, and multiphase wave 30% were observed in the right deltoid muscle. There was a >10% reduction in repetitive nerve stimulation (RNS) testing. The 3 and 5 Hz of paranasal muscles decreased by 16.3 and 13.9%, respectively. Mediastinum contrast-enhanced computed tomography (CT) showed plump lymph nodes, partial calcification, no abnormal density shadow, and no abnormal enhancement in the mediastinum. A left deltoid muscle biopsy demonstrated perivascular inflammation with necrosis, leading to the diagnosis of inflammatory myopathy (Figure 1).

Furthermore, the patient tested positive for AChR-Ab, titin-Ab, anti-M7, SMA-Ab, and CAE-Ab in immunoassays, while MuSK-Ab, RyR-Ab, and other myositis-associated autoantibodies were not detected (Tables 1, 2).

**Table 1:** Muscle disease related antibody list.

**Name**	**Test method**	**Results (titration)**	**Reference interval**
Anti- muscle antibody IgG	IIFT	+1:320	Negative
Anti-myocardial antibodyIgG	IIFT	+1:320	Negative
Anti-Titin antibody IgG	BLOT	++	Negative
Anti-SOX1 antibody IgG	BLOT	Negative	Negative
Anti-AChR antibodyIgG	ELISA	1.32 nmol/l positive	<0.4 nmol/l negative 0.4–0.5 nmol/lSuspicious >0.50 nmol/l positive
Anti-MuSK antibody IgG	ELISA	0.01 U/ml negative	≤ 0.4 U/ml negative >0.4 U/ml positive
RyR antibody IgG	ELISA	Negative	Negative
LRP-4antibody IgG	ELISA	Negative	Negative

**Table 2:** Inflammatory myopathy associated antibodies list.

**Name**	**Test method**	**Results (titration)**	**Reference interval**
Anti-Mi-2αantibody IgG	BLOT	Negative	Negative
Anti-TIF-1γantibody IgG	BLOT	Negative	Negative
Anti-NXP2antibody IgG	BLOT	Negative	Negative
Anti-Kuantibody IgG	BLOT	Negative	Negative
Anti-PM-ScI75antibody IgG	BLOT	Negative	Negative
Anti-SRPantibody IgG	BLOT	Negative	Negative
Anti-PL-12antibody IgG	BLOT	Negative	Negative
Anti-OJantibody IgG	BLOT	Negative	Negative
Anti-Mi-2βantibody IgG	BLOT	Negative	Negative
Anti-MDA5antibody IgG	BLOT	Negative	Negative
Anti-SAE1antibody IgG	BLOT	Negative	Negative
Anti-PM-Scl100antibody IgG	BLOT	Negative	Negative
Anti-Jo-1antibody IgG	BLOT	Negative	Negative
Anti-PL-7antibody IgG	BLOT	Negative	Negative
Anti-EJantibody IgG	BLOT	Negative	Negative
Anti-Ro-52antibody IgG	BLOT	Negative	Negative

The patient was diagnosed with concurrent MG and myositis and myocarditis, and was treated with intravenous immunoglobulin (IVIg) therapy (0.4 g/kg static ^*^5 d) and methylprednisolone (initially 80 mg/d, decreased to 60 mg/d after a week, and gradually reduced after 3 months). After treatment, significantly improvement in the patient's muscle strength, especially dysphagia, were noted. Repeated antibody tests in the follow-up 2 weeks showed reduced titers of AChR-Ab, titin-Ab anti-M7, SMA-Ab, and CAE-Ab.

## Discussion

Here, we report a rare case of concurrent myositis and myocarditis and MG with positive AChR-Ab and multiple muscle-related antibodies (titin, anti-M7, SMA, and CAE-Ab) in the absence of thymoma. At least 30 cases of concomitant manifestation of MG and IM have been reported previously (9). Among these patients, only four definitely confirmed cases were not accompanied by thymus lesions (10–13). In addition, only four cases of concurrent MG and both myositis and myocarditis (2, 14–16) as well as three cases of MG and myocarditis without myositis have been published (2, 17) to date. However, the absence of thymoma among these seven cases of the concomitant manifestation of MG and myocarditis has not been reported.

The thymus is a central immune organ and its dysfunction leads to many autoimmune diseases. Thymic malignancy is often related to paraneoplastic neurological diseases (PNDs). MG is the most common thymoma-associated PND, whereas disorders such as encephalitis and myositis are less common (18). Diagnosis of these disorders is often aided by testing for specific autoantibodies, including the well-characterized AChR-Ab for MG, although specific antibodies for MG and myositis are undetermined. Recent studies have shown that the presence of the striational antibodies, titin-Ab, and RyR-Ab, may indicate concurrent MG and myositis (8, 19) with up to 95% sensitivity and specificity for a thymoma in patients with MG; nevertheless, the role of these antibodies in the pathogenesis of these conditions has not yet been clearly elucidated (20). SMA-Ab and CAE-Ab, which are autoantibodies targeting skeletal muscle, are known to predict thymoma occurrence at an early stage (6, 7). Thus, to a certain degree, the occurrence of autoantibodies in MG and IM cases may also reflect thymic dysfunction. The absence of thymoma in the present case may be due to the undetectable size of the lesion by enhanced CT of the mediastinum. Alternatively, the PNDs may precede the tumor development, only becoming detectable in the follow-up.

The anti-mitochondrial antibody, anti-M7, has been shown to occur exclusively in the sera of patients with myocarditis or cardiomyopathies (4). However, the presence of anti-M7 in patients with MG and myocarditis, as well as its relationship with thymoma has not yet been reported. The possible pathogenic and diagnostic relevance of these situations requires further investigation.

Furthermore, with respect to the clinical manifestation, prominent bulbar symptoms, ptosis, diplopia, and fluctuating muscle fatigability should be regarded as the classic features of MG, as well as MG and myositis (21). Interestingly, our case did not have ophthalmoplegia, which suggested a distinct clinical presentation and the need for careful identification. Immunotherapy was also determined to be effective under these conditions.

In conclusion, we predict that the thymoma-associated autoantibodies (titin-Ab, SMA-Ab, and CAE-Ab) and anti-M7 may play an important role in concurrent of MG and myositis and myocarditis. Additional investigations are warranted to provide further evidence.

## Data Availability

The raw data supporting the conclusions of this manuscript will be made available by the authors, without undue reservation, to any qualified researcher.

## Author Contributions

ZZ and XC collected the clinical data and drafted the manuscript. JP and JW contributed to the clinical data collection and clinical management of the patients, and to the manuscript revision. GL performed skin biopsy analysis, created Figure 1, and contributed to the revision. All authors approved the final version of the manuscript.

### Conflict of Interest Statement

The authors declare that the research was conducted in the absence of any commercial or financial relationships that could be construed as a potential conflict of interest.
